# Aqua­bis(triphenyl­phosphine-κ*P*)copper(I) tetra­fluoridoborate

**DOI:** 10.1107/S1600536809029559

**Published:** 2009-07-29

**Authors:** Yanfeng Dai, Yi Zhang, Jianwen Tian, Zhen Liu

**Affiliations:** a Department of Chemistry, Nanchang University, Nanchang 330031, People’s Republic of China

## Abstract

In the title compound, [Cu(C_18_H_15_P)_2_(H_2_O)]BF_4_, the Cu^I^ atom is coordinated by two P atoms from triphenyl­phosphine ligands and one water mol­ecule in a distorted trigonal geometry. In the BF_4_^−^ anion, three F atoms are disordered over two sites around the B—F bond, the site-occupancy ratio being 0.67 (6):0.33 (6). The Cu⋯F distance of 2.602 (5) Å between the Cu atom and the ordered F atom may suggest a weak but genuine inter­action. O—H⋯F and weak C—H⋯F hydrogen bonding is present in the crystal structure.

## Related literature

For the applications of Cu^I^ complexes, see: Kirchhoff *et al.* (1985 ▶); Zhang *et al.* (2004 ▶); Moudam *et al.* (2007 ▶). For the tetra­hedral coordination geometry of Cu^I^ complexes, see: Engelhardt *et al.* (1985 ▶); Barron *et al.* (1987 ▶). For the weak Cu⋯F inter­action, see: Mao *et al.* (2003 ▶); Fu *et al.* (2004 ▶). For Cu—P and Cu—O bond distances, see: Meng *et al.* (2006 ▶).
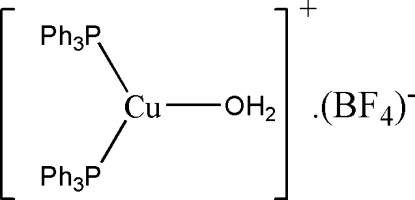


## Experimental

### 

#### Crystal data

[Cu(C_18_H_15_P)_2_(H_2_O)]BF_4_
*M* *_r_* = 692.91
Monoclinic, 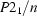

*a* = 13.9737 (14) Å
*b* = 12.4258 (11) Å
*c* = 19.4276 (18) Å
β = 94.521 (1)°
*V* = 3362.8 (5) Å^3^
*Z* = 4
Mo *K*α radiation
μ = 0.79 mm^−1^
*T* = 298 K
0.48 × 0.19 × 0.16 mm

#### Data collection

Bruker SMART APEXII area-detector diffractometer
Absorption correction: multi-scan (*SADABS*; Sheldrick, 1996 ▶) *T*_min_ = 0.702, *T*_max_ = 0.883
17192 measured reflections
5914 independent reflections
3008 reflections with *I* > 2σ(*I*)
*R*_int_ = 0.078

#### Refinement

*R*[*F* ^2^ > 2σ(*F* ^2^)] = 0.061
*wR*(*F* ^2^) = 0.202
*S* = 1.04
5914 reflections
434 parameters
1 restraint
H-atom parameters constrained
Δρ_max_ = 0.93 e Å^−3^
Δρ_min_ = −0.36 e Å^−3^

### 

Data collection: *APEX2* (Bruker, 2008 ▶); cell refinement: *SAINT* (Bruker, 2008 ▶); data reduction: *SAINT*; program(s) used to solve structure: *SHELXTL* (Sheldrick, 2008 ▶); program(s) used to refine structure: *SHELXTL*; molecular graphics: *SHELXTL*; software used to prepare material for publication: *SHELXTL*.

## Supplementary Material

Crystal structure: contains datablocks global, I. DOI: 10.1107/S1600536809029559/xu2555sup1.cif

Structure factors: contains datablocks I. DOI: 10.1107/S1600536809029559/xu2555Isup2.hkl

Additional supplementary materials:  crystallographic information; 3D view; checkCIF report

## Figures and Tables

**Table 1:** Selected bond lengths (Å)

Cu1—O1	2.105 (5)
Cu1—P1	2.2318 (18)
Cu1—P2	2.2478 (18)

**Table 2:** Hydrogen-bond geometry (Å, °)

*D*—H⋯*A*	*D*—H	H⋯*A*	*D*⋯*A*	*D*—H⋯*A*
O1—H1*C*⋯F2	0.85	1.87	2.71 (3)	171
O1—H1*D*⋯F3^i^	0.85	1.98	2.82 (3)	171
C28—H28⋯F4^ii^	0.93	2.51	3.25 (3)	137

Symmetry codes: (i) 
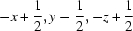
; (ii) 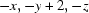.

**Fig. 1.**
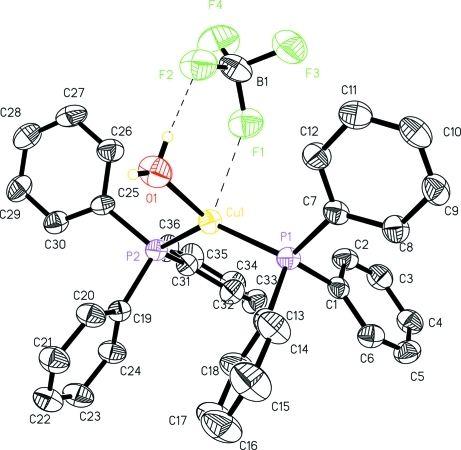
The molecular structure of the title compound. Displacement ellipsoids are drawn at the 30% probability level. The H atoms in benzene rings and the minor disorder component of the F2—F4 are omitted for clarity. The Cu···F weak interaction and O—H···F hydrogen bond are indicated by dashed lines.

## supplementary crystallographic information

### Comment

Copper(I) complexes with phosphine ligand have attracted much attention because of their rich photophysical properties and potential applications in organic light-emitting diodes (OLEDs) (Kirchhoff *et al.*, 1985; Zhang *et al.*, 2004; Moudam *et al.*, 2007). These complexes usually adopt tetrahedron coordination geometry (Engelhardt *et al.*, 1985; Barron *et al.*, 1987), three-coordinated copper(I) complexes with phosphine ligands is relatively little known. We reported here the title three-coordinated copper(I) complex.

The molecular structure is depicted in Fig. 1. The copper(I) atom is three-coordinated in distorted trigonal geometry (Table 1) by two P atoms from two triphenylphosphine ligands and one water molecule. The Cu1—P and Cu1—O bond distances are comparable to those found in related complexes (Engelhardt *et al.*, 1985; Barron *et al.*, 1987; Meng *et al.*, 2006). The coordination angles around the Cu1 atom are ranging from 104.80 (16)° to 133.89 (7)°. In the BF_4_ anion three F atoms are disordered over two sites around the B1—F1 bond. The Cu1···F1 distance of 2.602 (5) Å between the Cu1 atom and the ordered F1 atom may suggests a weak but genuine interaction, similar to the situation found in the related structures (Fu *et al.*, 2004); Mao *et al.*, 2003).

The O—H···F and weak C—H···F hydrogen bonding is present in the crystal structure (Table 2).

### Experimental

[Cu(CH_3_CN)_4_]BF_4_ (0.031 g, 0.1 mmol) was added to a solution of triphenylphosphine (0.052 g, 0.2 mmol) in 30 ml dichloromethane with small amount of water under nitrogen atmosphere. The mixture was stirred at room temperature for 2 h to obtain the yellow solution. Crystallization by slow diffusion of diethyl ether into the dichloromethane solution yielded yellow crystals suitable for X-ray diffraction (yield: 47%). Analysis calculated for [Cu(H_2_O)(C_18_H_15_P)_2_].(BF_4_): C 62.40, H 4.66%; Found: C 62.08, H 4.93%.

### Refinement

All H atoms were positioned geometrically and treated as riding (O—H = 0.65 Å and C—H = 0.93 Å, and refined in riding mode with *U*_iso_(H) = 1.2*U*_eq_(C,O). The F2, F3 and F4 atoms are disordered over two sites, site occupancy factors were refined to 0.67 (6) and 0.33 (6).

### Crystal data

**Table:**

[Cu(C_18_H_15_P)_2_(H_2_O)]BF_4_	*F*(000) = 1424
*M**_r_* = 692.91	*D*_x_ = 1.369 Mg m^−^^3^
Monoclinic, *P*2_1_/*n*	Mo *K*α radiation, λ = 0.71073 Å
Hall symbol: -P 2yn	Cell parameters from 2641 reflections
*a* = 13.9737 (14) Å	θ = 2.2–21.6°
*b* = 12.4258 (11) Å	µ = 0.79 mm^−^^1^
*c* = 19.4276 (18) Å	*T* = 298 K
β = 94.521 (1)°	Block, yellow
*V* = 3362.8 (5) Å^3^	0.48 × 0.19 × 0.16 mm
*Z* = 4	

### Data collection

**Table:**

Bruker SMART APEXII area-detector diffractometer	5914 independent reflections
Radiation source: fine-focus sealed tube	3008 reflections with *I* > 2σ(*I*)
graphite	*R*_int_ = 0.078
φ and ω scans	θ_max_ = 25.0°, θ_min_ = 1.7°
Absorption correction: multi-scan (*SADABS*; Sheldrick, 1996)	*h* = −16→15
*T*_min_ = 0.702, *T*_max_ = 0.883	*k* = −14→14
17192 measured reflections	*l* = −23→22

### Refinement

**Table:**

Refinement on *F*^2^	Primary atom site location: structure-invariant direct methods
Least-squares matrix: full	Secondary atom site location: difference Fourier map
*R*[*F*^2^ > 2σ(*F*^2^)] = 0.061	Hydrogen site location: inferred from neighbouring sites
*wR*(*F*^2^) = 0.202	H-atom parameters constrained
*S* = 1.03	*w* = 1/[σ^2^(*F*_o_^2^) + (0.0646*P*)^2^ + 7.3064*P*] where *P* = (*F*_o_^2^ + 2*F*_c_^2^)/3
5914 reflections	(Δ/σ)_max_ = 0.001
434 parameters	Δρ_max_ = 0.93 e Å^−^^3^
1 restraint	Δρ_min_ = −0.36 e Å^−^^3^

### Special details

**Table:**

Geometry. All e.s.d.'s (except the e.s.d. in the dihedral angle between two l.s. planes) are estimated using the full covariance matrix. The cell e.s.d.'s are taken into account individually in the estimation of e.s.d.'s in distances, angles and torsion angles; correlations between e.s.d.'s in cell parameters are only used when they are defined by crystal symmetry. An approximate (isotropic) treatment of cell e.s.d.'s is used for estimating e.s.d.'s involving l.s. planes.
Refinement. Refinement of *F*^2^ against ALL reflections. The weighted *R*-factor *wR* and goodness of fit *S* are based on *F*^2^, conventional *R*-factors *R* are based on *F*, with *F* set to zero for negative *F*^2^. The threshold expression of *F*^2^ > σ(*F*^2^) is used only for calculating *R*-factors(gt) *etc*. and is not relevant to the choice of reflections for refinement. *R*-factors based on *F*^2^ are statistically about twice as large as those based on *F*, and *R*- factors based on ALL data will be even larger.

### Fractional atomic coordinates and isotropic or equivalent isotropic displacement parameters (Å^2^)

**Table:**

	*x*	*y*	*z*	*U*_iso_*/*U*_eq_	Occ. (<1)
Cu1	0.36105 (6)	0.80395 (7)	0.13634 (4)	0.0517 (3)	
O1	0.2503 (4)	0.7985 (4)	0.2037 (2)	0.0875 (17)	
H1C	0.2344	0.8634	0.2107	0.105*	
H1D	0.2314	0.7597	0.2361	0.105*	
P1	0.50924 (12)	0.81613 (14)	0.18770 (8)	0.0460 (4)	
P2	0.29834 (12)	0.72605 (14)	0.03854 (8)	0.0464 (4)	
C1	0.6043 (4)	0.8457 (5)	0.1314 (3)	0.0463 (15)	
C2	0.5854 (5)	0.9201 (6)	0.0780 (3)	0.0606 (19)	
H2	0.5247	0.9510	0.0715	0.073*	
C3	0.6543 (6)	0.9483 (6)	0.0352 (4)	0.069 (2)	
H3	0.6399	0.9972	−0.0003	0.082*	
C4	0.7436 (6)	0.9051 (6)	0.0442 (4)	0.067 (2)	
H4	0.7902	0.9242	0.0149	0.081*	
C5	0.7649 (5)	0.8330 (6)	0.0971 (4)	0.068 (2)	
H5	0.8265	0.8044	0.1038	0.082*	
C6	0.6956 (5)	0.8025 (6)	0.1404 (3)	0.0583 (18)	
H6	0.7105	0.7530	0.1756	0.070*	
C7	0.5344 (5)	0.9112 (5)	0.2578 (3)	0.0512 (17)	
C8	0.6274 (5)	0.9386 (6)	0.2829 (3)	0.0610 (19)	
H8	0.6793	0.9041	0.2652	0.073*	
C9	0.6440 (6)	1.0154 (6)	0.3332 (4)	0.070 (2)	
H9	0.7066	1.0337	0.3487	0.084*	
C10	0.5692 (7)	1.0643 (7)	0.3602 (4)	0.077 (2)	
H10	0.5807	1.1151	0.3950	0.092*	
C11	0.4772 (6)	1.0403 (7)	0.3370 (4)	0.083 (3)	
H11	0.4262	1.0753	0.3554	0.100*	
C12	0.4599 (5)	0.9630 (6)	0.2857 (3)	0.067 (2)	
H12	0.3970	0.9462	0.2702	0.080*	
C13	0.5397 (5)	0.6851 (5)	0.2224 (3)	0.0527 (17)	
C14	0.5584 (6)	0.6645 (6)	0.2916 (4)	0.077 (2)	
H14	0.5589	0.7206	0.3234	0.092*	
C15	0.5764 (7)	0.5595 (8)	0.3144 (5)	0.105 (3)	
H15	0.5880	0.5459	0.3614	0.126*	
C16	0.5774 (8)	0.4772 (8)	0.2688 (5)	0.106 (3)	
H16	0.5910	0.4078	0.2845	0.127*	
C17	0.5585 (7)	0.4957 (7)	0.2005 (5)	0.094 (3)	
H17	0.5586	0.4392	0.1691	0.113*	
C18	0.5394 (6)	0.5986 (6)	0.1781 (4)	0.075 (2)	
H18	0.5257	0.6105	0.1311	0.090*	
C19	0.3094 (4)	0.5817 (5)	0.0445 (3)	0.0479 (16)	
C20	0.2846 (5)	0.5319 (6)	0.1046 (4)	0.067 (2)	
H20	0.2710	0.5738	0.1422	0.080*	
C21	0.2797 (6)	0.4224 (7)	0.1093 (4)	0.077 (2)	
H21	0.2616	0.3908	0.1496	0.092*	
C22	0.3010 (6)	0.3593 (7)	0.0560 (4)	0.077 (2)	
H22	0.2961	0.2849	0.0593	0.092*	
C23	0.3296 (6)	0.4054 (7)	−0.0029 (4)	0.081 (2)	
H23	0.3467	0.3624	−0.0392	0.097*	
C24	0.3332 (5)	0.5159 (6)	−0.0084 (4)	0.066 (2)	
H24	0.3521	0.5466	−0.0488	0.080*	
C25	0.1693 (5)	0.7425 (6)	0.0208 (3)	0.0487 (16)	
C26	0.1270 (5)	0.8370 (6)	0.0406 (4)	0.070 (2)	
H26	0.1638	0.8891	0.0645	0.084*	
C27	0.0280 (6)	0.8544 (8)	0.0245 (4)	0.083 (3)	
H27	−0.0007	0.9177	0.0380	0.099*	
C28	−0.0256 (6)	0.7771 (8)	−0.0113 (4)	0.079 (2)	
H28	−0.0904	0.7894	−0.0235	0.095*	
C29	0.0150 (5)	0.6832 (7)	−0.0291 (4)	0.073 (2)	
H29	−0.0223	0.6302	−0.0520	0.088*	
C30	0.1115 (5)	0.6663 (6)	−0.0133 (3)	0.0610 (19)	
H30	0.1386	0.6016	−0.0259	0.073*	
C31	0.3468 (5)	0.7605 (5)	−0.0425 (3)	0.0512 (17)	
C32	0.4439 (5)	0.7700 (6)	−0.0454 (4)	0.065 (2)	
H32	0.4840	0.7570	−0.0057	0.078*	
C33	0.4846 (6)	0.7984 (6)	−0.1059 (4)	0.074 (2)	
H33	0.5509	0.8030	−0.1069	0.089*	
C34	0.4255 (6)	0.8195 (7)	−0.1639 (4)	0.076 (2)	
H34	0.4519	0.8389	−0.2046	0.091*	
C35	0.3292 (6)	0.8125 (7)	−0.1625 (4)	0.080 (2)	
H35	0.2896	0.8278	−0.2021	0.096*	
C36	0.2894 (5)	0.7826 (6)	−0.1026 (3)	0.067 (2)	
H36	0.2231	0.7771	−0.1023	0.080*	
B1	0.2480 (10)	1.0703 (10)	0.1638 (6)	0.086 (3)	
F1	0.3118 (4)	1.0059 (4)	0.1338 (2)	0.0998 (15)	
F2	0.206 (2)	1.010 (2)	0.2129 (18)	0.108 (7)	0.67 (6)
F3	0.3076 (17)	1.150 (2)	0.1979 (18)	0.134 (9)	0.67 (6)
F4	0.188 (2)	1.115 (3)	0.1190 (10)	0.134 (10)	0.67 (6)
F2'	0.258 (5)	1.174 (2)	0.162 (3)	0.138 (17)	0.33 (6)
F3'	0.155 (3)	1.054 (5)	0.123 (3)	0.125 (15)	0.33 (6)
F4'	0.232 (5)	1.050 (6)	0.228 (2)	0.110 (15)	0.33 (6)

### Atomic displacement parameters (Å^2^)

**Table:**

	*U*^11^	*U*^22^	*U*^33^	*U*^12^	*U*^13^	*U*^23^
Cu1	0.0530 (5)	0.0605 (6)	0.0401 (5)	0.0008 (4)	−0.0054 (3)	−0.0045 (4)
O1	0.121 (4)	0.076 (4)	0.069 (3)	−0.003 (3)	0.034 (3)	0.015 (3)
P1	0.0515 (10)	0.0508 (11)	0.0342 (9)	0.0055 (9)	−0.0063 (7)	−0.0053 (8)
P2	0.0489 (10)	0.0510 (11)	0.0378 (9)	0.0044 (8)	−0.0063 (7)	−0.0019 (8)
C1	0.053 (4)	0.044 (4)	0.040 (4)	−0.001 (3)	−0.005 (3)	−0.009 (3)
C2	0.068 (5)	0.055 (5)	0.058 (4)	0.013 (4)	−0.003 (4)	0.000 (4)
C3	0.085 (6)	0.058 (5)	0.062 (5)	−0.002 (5)	0.004 (4)	0.008 (4)
C4	0.082 (6)	0.059 (5)	0.063 (5)	−0.002 (4)	0.016 (4)	−0.004 (4)
C5	0.062 (5)	0.075 (6)	0.069 (5)	0.012 (4)	0.009 (4)	−0.012 (4)
C6	0.066 (5)	0.059 (5)	0.050 (4)	0.010 (4)	0.000 (4)	0.003 (3)
C7	0.063 (4)	0.052 (4)	0.037 (4)	0.004 (4)	−0.005 (3)	−0.007 (3)
C8	0.067 (5)	0.067 (5)	0.048 (4)	0.001 (4)	−0.008 (3)	−0.011 (4)
C9	0.080 (5)	0.072 (6)	0.056 (5)	−0.007 (5)	−0.010 (4)	−0.012 (4)
C10	0.110 (7)	0.069 (6)	0.050 (5)	−0.006 (5)	0.002 (5)	−0.019 (4)
C11	0.089 (6)	0.089 (7)	0.074 (6)	0.004 (5)	0.022 (5)	−0.032 (5)
C12	0.070 (5)	0.075 (5)	0.055 (4)	0.002 (4)	0.002 (4)	−0.020 (4)
C13	0.059 (4)	0.055 (5)	0.042 (4)	−0.001 (4)	−0.006 (3)	−0.002 (3)
C14	0.108 (6)	0.060 (5)	0.058 (5)	−0.002 (5)	−0.016 (4)	0.003 (4)
C15	0.161 (10)	0.080 (7)	0.068 (6)	0.000 (7)	−0.028 (6)	0.024 (5)
C16	0.153 (9)	0.062 (6)	0.097 (8)	0.014 (6)	−0.023 (7)	0.016 (6)
C17	0.136 (8)	0.060 (6)	0.085 (7)	0.013 (6)	−0.007 (6)	−0.004 (5)
C18	0.105 (6)	0.059 (5)	0.059 (5)	0.016 (5)	−0.004 (4)	0.002 (4)
C19	0.050 (4)	0.053 (4)	0.040 (4)	0.006 (3)	−0.001 (3)	0.000 (3)
C20	0.088 (6)	0.057 (5)	0.057 (5)	0.009 (4)	0.014 (4)	0.000 (4)
C21	0.099 (6)	0.064 (6)	0.069 (5)	0.007 (5)	0.016 (5)	0.011 (4)
C22	0.096 (6)	0.054 (5)	0.080 (6)	0.010 (5)	0.002 (5)	0.004 (5)
C23	0.107 (7)	0.062 (6)	0.073 (6)	0.014 (5)	0.013 (5)	−0.010 (4)
C24	0.084 (5)	0.060 (5)	0.056 (5)	0.011 (4)	0.009 (4)	0.001 (4)
C25	0.052 (4)	0.054 (4)	0.040 (4)	0.013 (3)	0.002 (3)	0.004 (3)
C26	0.070 (5)	0.066 (5)	0.072 (5)	0.013 (4)	−0.001 (4)	0.000 (4)
C27	0.077 (6)	0.082 (6)	0.091 (6)	0.035 (5)	0.014 (5)	0.012 (5)
C28	0.060 (5)	0.106 (8)	0.071 (6)	0.017 (5)	−0.004 (4)	0.009 (5)
C29	0.052 (4)	0.102 (7)	0.063 (5)	0.002 (5)	−0.007 (4)	−0.007 (5)
C30	0.054 (4)	0.074 (5)	0.053 (4)	0.009 (4)	−0.006 (3)	−0.007 (4)
C31	0.057 (4)	0.054 (4)	0.042 (4)	0.000 (3)	−0.002 (3)	0.002 (3)
C32	0.063 (5)	0.075 (5)	0.056 (5)	0.003 (4)	0.002 (4)	0.009 (4)
C33	0.067 (5)	0.081 (6)	0.075 (5)	0.004 (4)	0.019 (4)	0.013 (5)
C34	0.091 (6)	0.081 (6)	0.058 (5)	−0.002 (5)	0.018 (5)	0.008 (4)
C35	0.088 (6)	0.099 (7)	0.051 (5)	−0.011 (5)	−0.005 (4)	0.018 (4)
C36	0.063 (5)	0.086 (6)	0.050 (4)	−0.009 (4)	−0.001 (4)	0.012 (4)
B1	0.123 (10)	0.058 (8)	0.075 (8)	0.007 (8)	0.003 (8)	0.002 (6)
F1	0.111 (4)	0.107 (4)	0.085 (3)	0.013 (3)	0.026 (3)	0.007 (3)
F2	0.131 (14)	0.094 (13)	0.108 (16)	0.015 (9)	0.058 (12)	0.023 (9)
F3	0.134 (12)	0.113 (11)	0.151 (18)	−0.001 (10)	−0.009 (11)	−0.046 (11)
F4	0.139 (14)	0.14 (2)	0.119 (9)	0.048 (15)	−0.014 (9)	0.047 (13)
F2'	0.18 (4)	0.097 (19)	0.14 (3)	−0.014 (19)	−0.01 (3)	0.015 (17)
F3'	0.14 (2)	0.10 (3)	0.13 (2)	0.02 (2)	−0.016 (18)	−0.024 (19)
F4'	0.15 (3)	0.12 (4)	0.060 (15)	0.02 (3)	0.013 (16)	0.013 (18)

### Geometric parameters (Å, °)

**Table:**

Cu1—O1	2.105 (5)	C17—H17	0.9300
Cu1—P1	2.2318 (18)	C18—H18	0.9300
Cu1—P2	2.2478 (18)	C19—C24	1.375 (9)
O1—H1C	0.8500	C19—C20	1.389 (9)
O1—H1D	0.8500	C20—C21	1.366 (10)
P1—C13	1.800 (7)	C20—H20	0.9300
P1—C7	1.816 (6)	C21—C22	1.349 (10)
P1—C1	1.823 (6)	C21—H21	0.9300
P2—C19	1.803 (7)	C22—C23	1.368 (10)
P2—C31	1.814 (6)	C22—H22	0.9300
P2—C25	1.820 (6)	C23—C24	1.377 (10)
C1—C6	1.383 (8)	C23—H23	0.9300
C1—C2	1.400 (9)	C24—H24	0.9300
C2—C3	1.366 (9)	C25—C30	1.380 (9)
C2—H2	0.9300	C25—C26	1.382 (9)
C3—C4	1.357 (10)	C26—C27	1.410 (10)
C3—H3	0.9300	C26—H26	0.9300
C4—C5	1.378 (10)	C27—C28	1.373 (11)
C4—H4	0.9300	C27—H27	0.9300
C5—C6	1.385 (9)	C28—C29	1.355 (11)
C5—H5	0.9300	C28—H28	0.9300
C6—H6	0.9300	C29—C30	1.375 (9)
C7—C12	1.373 (9)	C29—H29	0.9300
C7—C8	1.394 (9)	C30—H30	0.9300
C8—C9	1.374 (9)	C31—C32	1.367 (9)
C8—H8	0.9300	C31—C36	1.390 (9)
C9—C10	1.350 (10)	C32—C33	1.391 (9)
C9—H9	0.9300	C32—H32	0.9300
C10—C11	1.362 (10)	C33—C34	1.369 (10)
C10—H10	0.9300	C33—H33	0.9300
C11—C12	1.391 (10)	C34—C35	1.351 (10)
C11—H11	0.9300	C34—H34	0.9300
C12—H12	0.9300	C35—C36	1.381 (9)
C13—C14	1.374 (9)	C35—H35	0.9300
C13—C18	1.377 (9)	C36—H36	0.9300
C14—C15	1.393 (11)	B1—F4	1.285 (19)
C14—H14	0.9300	B1—F2'	1.30 (3)
C15—C16	1.354 (12)	B1—F4'	1.31 (5)
C15—H15	0.9300	B1—F1	1.363 (12)
C16—C17	1.352 (11)	B1—F2	1.38 (3)
C16—H16	0.9300	B1—F3	1.42 (2)
C17—C18	1.370 (10)	B1—F3'	1.48 (4)
			
O1—Cu1—P1	115.18 (16)	C17—C18—C13	122.6 (7)
O1—Cu1—P2	104.80 (16)	C17—C18—H18	118.7
P1—Cu1—P2	133.89 (7)	C13—C18—H18	118.7
Cu1—O1—H1C	106.5	C24—C19—C20	117.0 (7)
Cu1—O1—H1D	139.8	C24—C19—P2	124.6 (5)
H1C—O1—H1D	108.6	C20—C19—P2	118.1 (5)
C13—P1—C7	106.4 (3)	C21—C20—C19	121.1 (7)
C13—P1—C1	104.2 (3)	C21—C20—H20	119.4
C7—P1—C1	102.2 (3)	C19—C20—H20	119.4
C13—P1—Cu1	106.8 (2)	C22—C21—C20	120.8 (8)
C7—P1—Cu1	119.8 (2)	C22—C21—H21	119.6
C1—P1—Cu1	116.1 (2)	C20—C21—H21	119.6
C19—P2—C31	104.8 (3)	C21—C22—C23	119.7 (8)
C19—P2—C25	101.7 (3)	C21—C22—H22	120.2
C31—P2—C25	103.9 (3)	C23—C22—H22	120.2
C19—P2—Cu1	110.4 (2)	C22—C23—C24	119.8 (8)
C31—P2—Cu1	119.0 (2)	C22—C23—H23	120.1
C25—P2—Cu1	115.2 (2)	C24—C23—H23	120.1
C6—C1—C2	118.0 (6)	C19—C24—C23	121.5 (7)
C6—C1—P1	123.7 (5)	C19—C24—H24	119.2
C2—C1—P1	118.3 (5)	C23—C24—H24	119.2
C3—C2—C1	121.3 (7)	C30—C25—C26	118.0 (6)
C3—C2—H2	119.4	C30—C25—P2	123.2 (5)
C1—C2—H2	119.4	C26—C25—P2	118.8 (6)
C4—C3—C2	120.3 (7)	C25—C26—C27	120.2 (8)
C4—C3—H3	119.8	C25—C26—H26	119.9
C2—C3—H3	119.8	C27—C26—H26	119.9
C3—C4—C5	119.8 (7)	C28—C27—C26	119.4 (8)
C3—C4—H4	120.1	C28—C27—H27	120.3
C5—C4—H4	120.1	C26—C27—H27	120.3
C4—C5—C6	120.7 (7)	C29—C28—C27	120.6 (8)
C4—C5—H5	119.7	C29—C28—H28	119.7
C6—C5—H5	119.7	C27—C28—H28	119.7
C1—C6—C5	119.9 (6)	C28—C29—C30	119.8 (8)
C1—C6—H6	120.0	C28—C29—H29	120.1
C5—C6—H6	120.0	C30—C29—H29	120.1
C12—C7—C8	117.6 (6)	C29—C30—C25	121.9 (7)
C12—C7—P1	119.6 (5)	C29—C30—H30	119.0
C8—C7—P1	122.8 (5)	C25—C30—H30	119.0
C9—C8—C7	121.3 (7)	C32—C31—C36	117.2 (6)
C9—C8—H8	119.4	C32—C31—P2	119.7 (5)
C7—C8—H8	119.4	C36—C31—P2	123.0 (5)
C10—C9—C8	119.8 (7)	C31—C32—C33	122.0 (7)
C10—C9—H9	120.1	C31—C32—H32	119.0
C8—C9—H9	120.1	C33—C32—H32	119.0
C9—C10—C11	120.9 (7)	C34—C33—C32	118.9 (7)
C9—C10—H10	119.6	C34—C33—H33	120.5
C11—C10—H10	119.6	C32—C33—H33	120.5
C10—C11—C12	119.6 (7)	C35—C34—C33	120.6 (7)
C10—C11—H11	120.2	C35—C34—H34	119.7
C12—C11—H11	120.2	C33—C34—H34	119.7
C7—C12—C11	120.8 (7)	C34—C35—C36	120.1 (7)
C7—C12—H12	119.6	C34—C35—H35	120.0
C11—C12—H12	119.6	C36—C35—H35	120.0
C14—C13—C18	117.2 (7)	C35—C36—C31	121.2 (7)
C14—C13—P1	123.8 (6)	C35—C36—H36	119.4
C18—C13—P1	118.9 (5)	C31—C36—H36	119.4
C13—C14—C15	120.0 (8)	F4—B1—F1	112.3 (13)
C13—C14—H14	120.0	F4—B1—F2	114.1 (18)
C15—C14—H14	120.0	F1—B1—F2	107.7 (14)
C16—C15—C14	120.7 (8)	F4—B1—F3	110.3 (14)
C16—C15—H15	119.6	F1—B1—F3	103.1 (12)
C14—C15—H15	119.6	F2—B1—F3	108.8 (13)
C17—C16—C15	120.2 (9)	F2'—B1—F4'	104 (3)
C17—C16—H16	119.9	F2'—B1—F3'	103 (2)
C15—C16—H16	119.9	F4'—B1—F3'	106 (3)
C16—C17—C18	119.2 (8)	F1—B1—F3'	105.6 (16)
C16—C17—H17	120.4	F2'—B1—F1	120 (2)
C18—C17—H17	120.4	F4'—B1—F1	117 (3)
			
O1—Cu1—P1—C13	75.2 (3)	C13—C14—C15—C16	1.1 (15)
P2—Cu1—P1—C13	−72.4 (2)	C14—C15—C16—C17	−1.5 (17)
O1—Cu1—P1—C7	−45.7 (3)	C15—C16—C17—C18	0.6 (17)
P2—Cu1—P1—C7	166.8 (2)	C16—C17—C18—C13	0.8 (15)
O1—Cu1—P1—C1	−169.2 (3)	C14—C13—C18—C17	−1.3 (12)
P2—Cu1—P1—C1	43.3 (3)	P1—C13—C18—C17	−177.8 (7)
O1—Cu1—P2—C19	−83.8 (3)	C31—P2—C19—C24	−10.6 (7)
P1—Cu1—P2—C19	66.0 (2)	C25—P2—C19—C24	97.4 (6)
O1—Cu1—P2—C31	155.1 (3)	Cu1—P2—C19—C24	−139.8 (5)
P1—Cu1—P2—C31	−55.1 (3)	C31—P2—C19—C20	175.1 (5)
O1—Cu1—P2—C25	30.6 (3)	C25—P2—C19—C20	−76.9 (6)
P1—Cu1—P2—C25	−179.5 (2)	Cu1—P2—C19—C20	45.8 (6)
C13—P1—C1—C6	−26.4 (6)	C24—C19—C20—C21	−3.0 (11)
C7—P1—C1—C6	84.2 (6)	P2—C19—C20—C21	171.7 (6)
Cu1—P1—C1—C6	−143.5 (5)	C19—C20—C21—C22	1.4 (13)
C13—P1—C1—C2	156.7 (5)	C20—C21—C22—C23	1.4 (13)
C7—P1—C1—C2	−92.7 (5)	C21—C22—C23—C24	−2.4 (13)
Cu1—P1—C1—C2	39.6 (6)	C20—C19—C24—C23	2.0 (11)
C6—C1—C2—C3	1.0 (10)	P2—C19—C24—C23	−172.4 (6)
P1—C1—C2—C3	178.1 (6)	C22—C23—C24—C19	0.7 (12)
C1—C2—C3—C4	−0.8 (11)	C19—P2—C25—C30	−28.7 (6)
C2—C3—C4—C5	−0.3 (12)	C31—P2—C25—C30	80.0 (6)
C3—C4—C5—C6	1.1 (11)	Cu1—P2—C25—C30	−148.0 (5)
C2—C1—C6—C5	−0.2 (10)	C19—P2—C25—C26	153.1 (5)
P1—C1—C6—C5	−177.1 (5)	C31—P2—C25—C26	−98.3 (6)
C4—C5—C6—C1	−0.8 (11)	Cu1—P2—C25—C26	33.7 (6)
C13—P1—C7—C12	−111.1 (6)	C30—C25—C26—C27	−1.5 (10)
C1—P1—C7—C12	139.9 (6)	P2—C25—C26—C27	176.8 (6)
Cu1—P1—C7—C12	10.0 (7)	C25—C26—C27—C28	−0.4 (12)
C13—P1—C7—C8	72.0 (6)	C26—C27—C28—C29	2.3 (13)
C1—P1—C7—C8	−37.0 (6)	C27—C28—C29—C30	−2.2 (12)
Cu1—P1—C7—C8	−166.9 (5)	C28—C29—C30—C25	0.2 (11)
C12—C7—C8—C9	−0.7 (10)	C26—C25—C30—C29	1.6 (10)
P1—C7—C8—C9	176.2 (6)	P2—C25—C30—C29	−176.6 (5)
C7—C8—C9—C10	1.3 (11)	C19—P2—C31—C32	−81.9 (6)
C8—C9—C10—C11	−1.5 (13)	C25—P2—C31—C32	171.8 (6)
C9—C10—C11—C12	1.1 (13)	Cu1—P2—C31—C32	42.0 (7)
C8—C7—C12—C11	0.3 (11)	C19—P2—C31—C36	100.8 (6)
P1—C7—C12—C11	−176.7 (6)	C25—P2—C31—C36	−5.5 (7)
C10—C11—C12—C7	−0.5 (13)	Cu1—P2—C31—C36	−135.3 (6)
C7—P1—C13—C14	12.0 (7)	C36—C31—C32—C33	−1.2 (11)
C1—P1—C13—C14	119.6 (6)	P2—C31—C32—C33	−178.7 (6)
Cu1—P1—C13—C14	−117.1 (6)	C31—C32—C33—C34	1.3 (12)
C7—P1—C13—C18	−171.7 (6)	C32—C33—C34—C35	−0.3 (13)
C1—P1—C13—C18	−64.2 (6)	C33—C34—C35—C36	−0.7 (13)
Cu1—P1—C13—C18	59.2 (6)	C34—C35—C36—C31	0.8 (13)
C18—C13—C14—C15	0.3 (12)	C32—C31—C36—C35	0.1 (11)
P1—C13—C14—C15	176.6 (7)	P2—C31—C36—C35	177.5 (6)

### Hydrogen-bond geometry (Å, °)

**Table:**

*D*—H···*A*	*D*—H	H···*A*	*D*···*A*	*D*—H···*A*
O1—H1C···F2	0.85	1.87	2.71 (3)	171
O1—H1D···F3^i^	0.85	1.98	2.82 (3)	171
C28—H28···F4^ii^	0.93	2.51	3.25 (3)	137

Symmetry codes: (i) −*x*+1/2, *y*−1/2, −*z*+1/2; (ii) −*x*, −*y*+2, −*z*.
